# Gender Differences in the Life Course Effects of Unemployment on Mid- and Later-Life Health

**DOI:** 10.1093/geroni/igaa057.1952

**Published:** 2020-12-16

**Authors:** Amal Harrati, Peter Heburn

**Affiliations:** 1 Stanford University, Berkeley, California, United States; 2 Princeton University, Princeton, New Jersey, United States

## Abstract

There is substantial evidence that unemployment is associated with adverse health. Given different lifetime employment patterns, these effects may differ between men and women. However, current studies often only characterize unemployment as a one-time shock, and measure the effects on health shortly thereafter. Using unique data available from The National Longitudinal Study of Youth 1979, we characterize employment trajectories for a nationally-representative sample of American men and women for every week of their lives between the ages of 18 and 50 years old. We then explore associations between unemployment and a number of health conditions including cancer, hypertension, diabetes, and depression at age 50--when the onset of chronic health conditions often begins—to examine the cumulative effects of unemployment over the life course on later-life health. We find that men and women have different patterns of lifetime unemployment and that these patterns have strong associations with poorer health at age 50.

